# The impact of dietary interventions on quality of life in adults with inflammatory bowel disease: a narrative review

**DOI:** 10.1017/jns.2026.10096

**Published:** 2026-04-24

**Authors:** Rebekah McGuire, Linda L. Knol, Joy Douglas

**Affiliations:** Human Nutrition, The University of Alabama, USA

**Keywords:** Crohn’s disease, Dietary intervention, Dietary recommendations, Inflammatory bowel disease, Quality of life, Ulcerative colitis

## Abstract

Symptoms associated with chronic inflammation have a negative impact on quality of life (QOL) in adults with inflammatory bowel disease (IBD). Dietary changes may assist in improving symptoms; however, they can be difficult to implement, causing increased stress and decreased QOL. The purpose of this narrative review was to investigate the impact of dietary interventions on QOL in adults with IBD. EBSCOhost was used to simultaneously search eight databases using the search terms ‘inflammatory bowel disease’ or ‘IBD’ or ‘ulcerative colitis’ or ‘Crohn’s disease’ AND ‘dietary interventions’ or ‘dietary advice’ or ‘dietary recommendations’. Inclusion criteria included adults with IBD and a validated QOL measure. After screening 1054 studies, 15 clinical trials were identified. Among these studies, dietary interventions included the low fermentable oligosaccharides, disaccharides, monosaccharides, and polyols (FODMAP) diet (n = 4), Immunoglobulin G (IgG) diet (n = 2), Anti-Inflammatory Diet (AID) (n = 1), highly restricted organic diet (n = 1), Autoimmune Protocol Diet (AIP) (n = 1), Dietary Modified Program (DMP) (n = 1), Dietary Modified Framework (DMF) (n = 1), Mediterranean diet (n = 2), and a high fibre diet (n = 2). QOL was measured using various validated questionnaires. Significant improvements in QOL were found in two of the low FODMAP diet studies, both IgG diet studies, the DMP, and one high fibre diet study. The Anti-inflammatory Diet, Dietary Modified Framework, Autoimmune Protocol Diet, highly restricted organic diet, and Mediterranean diet did not significantly improve QOL. Future research should focus on comparing dietary interventions, longer study durations, diet adherence and QOL. Due to the complexity in the various diets, dietitians with expertise in IBD are needed to assist with diet management.

## Graphical abstract

**Figure:**
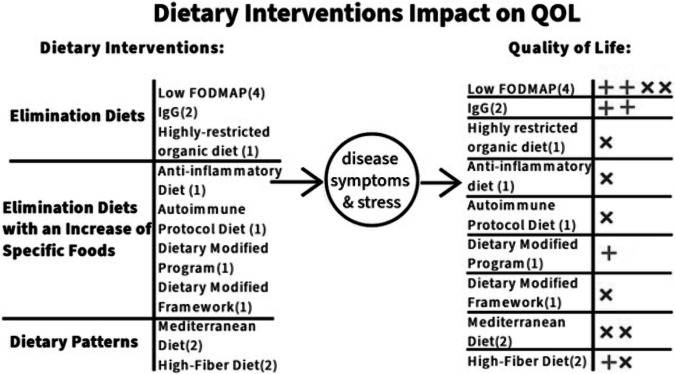

## Introduction

According to the Global Burden of Disease 2021, there were approximately 3.8 million cases of inflammatory bowel disease (IBD) worldwide.^(1)^ IBD, an autoimmune disease, causes chronic inflammation in the gastrointestinal (GI) tract.^(2,3)^ IBD, including Crohn’s disease (CD) can occur as inflammatory patches anywhere between the mouth and anus, while ulcerative colitis (UC) is localized to the large intestine. Additionally, CD penetrates through many layers of the GI tract, whereas UC is localized to the surface of the intestinal wall. Tools such as the Mayo Score, for UC, and the Crohn’s Disease Activity Index (CDAI) and the Harvey-Bradshaw Index (HBI), for CD, are used to measure disease severity based on individual disease characteristics and symptoms (Table 1).^(4–6)^ IBD symptoms can include abdominal pain and cramping, frequent urgent bowel movements, blood or mucus in stool, and non-GI related symptoms such as joint pain and fatigue, all of which can impact quality of life (QOL).^(7)^ Individuals with IBD can experience intermittent ranges of disease activity from remission with very mild or no symptoms to a flare, which occurs when symptoms and inflammation are severe. Changes in diet can potentially decrease symptoms and therefore increase QOL for these patients; however, the current dietary approaches may be difficult to manage or lead to stress and feelings of deprivation and therefore decrease QOL.^(8)^ Many patients with IBD request diet information from providers.^(9)^ However, dietary advice varies for this population, likely reflecting a lack of evidence-based dietary guidelines for IBD.^(10)^

**Table 1.** Measures of disease severity utilized in the included studies

Measure	IBD type	Questions	Reference period	Scores
Crohn’s Disease Activity Index (CDAI)^6^	CD only	Weight, sex, signs and symptoms	7 days	<150 represents remission. > 450 represents severe disease.
Harvey-Bradshaw Index^4^	CD only	General well-being, Signs and symptoms	Previous day	<5 represents remission. 5–7 represents mild disease. 8–16 represents moderate disease. >16 represents severe disease.
Mayo score^5^	UC only	Bowel movements and disease activity along with endoscopic appearance	N/A	0–2 represents remission. 3–5 represents mild disease. 6–10 represents moderate disease activity. 11–12 represents severe.
Partial Mayo score^32^	UC only	Bowel movements and disease activity but does not consider endoscopic appearance	N/A	<2 represents remission. 2–4 represents mild disease. 5–7 represents moderate disease activity. 7–9 represents severe disease activity.
Simple Clinical Colitis Index	UC only	Bowel movements, well-being, and disease symptoms	3 days	The score ranges from 0–19 with a higher score indicating more severe disease.

CD, Crohn’s Disease.

UC, Ulcerative colitis.

Previous reviews have evaluated dietary interventions in adults with IBD, but the reporting of QOL within these reviews was not consistent. For example, four systematic reviews have explored the impact of dietary interventions on IBD.^(10–13)^ In two of the reviews, by Limketkai et al.^(10)^ and Nieva et al.^(11)^ QOL was not the primary outcome of interest and was therefore missing from many included studies. Similarly, Abbas et al.^(12)^ included studies with QOL as an outcome measure; however, they did not include both IBD types. Lastly, Gomes et al.^(13)^ did not include QOL as an outcome measure. QOL can be negatively impacted by a variety of factors associated with IBD such as symptoms and treatments, thus it is important to consider for this population. As such, there is a further need to focus on QOL as the main outcome. Therefore, the purpose of this review is to explore clinical trials that focus on the impact of dietary interventions on disease specific, health-related QOL in adults with IBD.

## Methods and materials

### Study inclusion and exclusion criteria

Study inclusion criteria was based on study design, dietary pattern interventions, and outcomes. Randomized control trials and quasi-experimental studies with dietary pattern interventions that involved individuals with CD or UC, regardless of sex or ethnicity, were considered for review. Studies must have measured QOL using a health-related QOL questionnaire as an outcome. Time and language restrictions were not applied to these searches. Studies were excluded if the population consisted of children or adolescents under the age of 18 or had a nutrition support intervention, such as enteral or parenteral nutrition.

### Literature search methods

This narrative review was developed using multiple databases to identify full-text, peer-reviewed articles. The EBSCOhost platform was utilized with eight chosen databases: Academic Search Premier, ALT Health Watch, Cochrane Central Register of Controlled Trials, Cochrane Database of Systematic Reviews, Health Source: Nursing/Academic Edition, CINAHL Ultimate, Cochrane Clinical Answers, and MEDLINE. Boolean search terms included ‘inflammatory bowel disease’ or ‘IBD’ or ‘ulcerative colitis’ or ‘Crohn’s disease’ AND ‘dietary interventions’ or ‘dietary advice’ or ‘dietary recommendations’. This search, completed in October 2024, resulted in 1054 total articles with 222 duplicates removed. The remaining 832 articles were screened for titles meeting inclusion criteria. Of these, 67 articles were eligible for retrieval. After reviewing abstracts, 24 articles met the eligibility for full text retrieval and screening. Seven of the 24 articles were eligible for this review based on the inclusion and exclusion criteria. Additionally, the PubMed database was searched using the original Boolean terms previously listed. PubMed was also set to filter for randomized controlled trials and clinical trials only, which resulted in 9 total studies; however, this did not add any new articles for this review. Lastly, hand searches of reference lists were completed and eight additional articles met inclusion criteria for a total of 15 articles, representing 15 studies to be included in this review. Titles and abstracts were screened and chosen by the first author (RM). Full text articles were then read and chosen for data extraction by the first author (RM). Information extracted included study design, participant demographics, IBD type, study duration, description of the intervention and control group diets, QOL measures, and changes in QOL. The second author (LK) then screened full text articles for the previously mentioned study criteria. Discussion between the authors (RM and LK) took place to resolve any disagreements in study eligibility or data extraction. Any conflicts were resolved after discussion with the third author (JD).

## Results

### Common dietary approaches used to reduce symptoms of IBD

Selected studies include a variety of dietary approaches. The results will be grouped based on dietary intervention similarities (Table 2). For example, seven of the research teams used dietary approaches that focused on eliminating foods from the diet, such as the low FODMAP diet, a highly restricted organic diet, and the immunoglobulin-G (IgG) diet, which requires testing an individual’s serum for a response of IgG antibodies after exposure to certain food items.^(14–20)^ Other approaches, such as the Anti-Inflammatory Diet (AID), Autoimmune Protocol Diet (AIP), Dietary Modification Framework (DMF), and Dietary Modification Program (DMP) focused on eliminating specific foods associated with worsening symptoms while increasing intake of foods that may reduce GI inflammation, symptoms, and dysbiosis.^(21–24)^ Although vegetarian or vegan dietary patterns were not identified specifically, the last group of dietary approaches used focused on specific foods or food groups that may reduce inflammation such as the Mediterranean diet (MD) and high fibre diets that focus on including foods or food groups that promote intake of anti-inflammatory foods, such as fruits and vegetables, and improved gut health.^(25–28)^ Refer to Table 2 for descriptions of these diets.

**Table 2.** Description of dietary approaches used across included studies

Diet	Foods to eliminate	Foods to include	Procedures
Low FODMAP^14,16–18^	Eliminate all high FODMAP foods over 2–6 weeks, including artichoke, asparagus, cauliflower, garlic, green peas, mushrooms, onion, sugar snap peas, apples, apple juice, cherries, dried fruit, mango, nectarines, peaches, pears, plums, watermelon, cow’s milk, custard, evaporated milk, ice cream, soy milk, sweetened condensed milk, yogurt, legumes, marinated and processed meats, breads, breakfast cereals, biscuits and snack products with wheat/rye/barley, high fructose corn syrup, honey, sugar free confectionery, cashews, pistachios		Reintroduce high FODMAP foods one at a time over 8–12 weeks while monitoring for symptoms. Maintain a long-term intake of tolerated foods.
IgG^19^	Egg, wheat, milk, corn, tomato, crab, rice, soybean, cod, shrimp, mushroom, beef, chicken, pork		Remove foods with IgG antibody titres of more than 100 U/mL and limit foods with IgG antibody titres of 50–100 U/mL for six months.
IgG4^15^	Milk, milk casein, peanuts, soy, shrimp, whole egg, tomato, pork, beef, cod, potato, wheat, yeast, cheddar cheese, chicken, lamb, rice		Exclude the four foods that had the highest titre response.
Highly restricted, organic diet^20^	Eliminate foods that were not from highly monitored organic farming including fruits and vegetables.	Red meat cooked in rapeseed oil (<2 T per day), sourdough bread made from a specific Viennese bakery with fresh butter (<20 g per day). Plain tap water and organic tea. Rock salt.	Consume organic farmed foods only.
Anti-inflammatory Diet^24^	Red and processed meats, added sugars	Increase intake of dietary fibres and prebiotic foods such as legumes, onion, garlic, and asparagus, probiotic foods such as yogurt, and omega-3 PFAs from 2 services of fish, specifically salmon, per week	Consume foods that are high in anti-oxidants and have been shown to improved IBD-related symptoms.
Autoimmune Protocol Diet^21^	Eliminate grains, legumes, nightshades, eggs, dairy, nuts and seeds, coffee, alcohol, refined/processed sugars, industrial seed oils, food additives, and the use of nonsteroidal anti-inflammatory drugs until no longer experiencing symptoms.	They may consume fresh foods that are nutrient-dense, probiotics and fermented foods, and bone broth. Other lifestyle factors such as physical activity, stress management, and adequate sleep are emphasized during this phase.	Eliminated foods can be reintroduced once symptoms subside.
Specific Carbohydrate Diet^25^	Eliminate certain starchy vegetables such as potatoes and yams, any canned fruits and vegetables, legumes such as chickpeas and soybeans, all grains, processed/canned/smoked meats, and dairy.	Include fruits and vegetables, legumes such as lentils and split peas, unprocessed meats, some cheeses, and homemade yogurt with limited lactose. Saccharin and honey were allowed as sweeteners.	
Dietary Modification Program^23^	Low FODMAP diet in addition to reducing fat, simple carbohydrates, and eliminating dairy, alcohol, and caffeine.	Focus on increasing quality protein and probiotic foods.	
Dietary Modified Framework^22^	Low FODMAP, low fat, low fibre	Encouraged high intake of protein and probiotics	
Mediterranean Diet^25,28^		Include fruits, vegetables, nuts, fish, whole grains, and olive oils	
High-fibre Diet^26^	Low refined carbohydrate	High fibre foods including ½ cup of whole wheat bran cereal, and 48 oz of fluids per day.	
Low-fat, high-fibre diet^27^		10% of calories from fat, 1–5% from saturated fat, and 5–9% from unsaturated fat (omega 6:3 ratio of 3:1 or lower). Increase in overall fibre intake from fruits, vegetables, and whole grains.	

FODMAP, fermentable oligosaccharides, disaccharides, monosaccharides, and polyols.

IgG, Immunoglobulin G.

### Measures of quality of life use in studies

Each study met inclusion criteria by exploring QOL as either a primary or secondary outcome measure. This was observed using validated QOL measures, most of which were specific to IBD (Table 3). For example, the Inflammatory Bowel Disease Questionnaire (IBDQ) is a validated, 32-item questionnaire that assesses the disease-related QOL for individuals with IBD^(29)^ and was used most frequently within these studies.^(14,18,19,20,22,26,28)^ The short IBDQ (sIBDQ).^(30)^ is a shortened version of the IBDQ (containing 10 questions) and was utilized in six of the included studies.^(15,17,21,24,25,27)^ Additionally, the Short Form 36 Health Survey (SF-36)^(31)^ is a validated questionnaire that assesses health-related QOL, though not specific to individuals with IBD, and was used in three studies.^(16,23,27)^

**Table 3.** Measures of QOL utilized across included studies

Name	Number of items	Domains	Scoring
Inflammatory Bowel Disease Questionnaire (IBD-Q)^29^	32-items	Bowel symptoms, Systemic symptoms, Emotional function, and Social function	0 to 224 where an increase in score indicates an increase in QOL
Short-IBDQ (SIBDQ)^30^	10-items	Bowel symptoms, Systemic symptoms, Emotional function, and social function	1 to 7 where an increase in score indicates an increase in QOL.
Short Form-36 (SF-36)^31^	36-items	Physical functioning, Bodily pain, role limitations due to physical health problems, Role limitations due to personal or emotional problems, Emotional well-being, Social functioning, Energy/fatigue, and General health perceptions.	0 to 100, where an increase in score indicates an increase in QOL.

IBDQ, Inflammatory Bowel Disease Questionnaire.

QOL, quality of life.

### Study design, setting, and duration

Of the 15 studies selected, 13 were randomized controlled trials (RCT).^(14–20,22–27)^ Six studies were unblinded,^(17,19,22,24,25,27)^ whereas three were double-blinded,^(15,16,20)^ three were single-blinded,^(18,23,26)^ and two were cross-over studies.^(16,27)^ In addition, two studies were uncontrolled trials.^(21,28)^ These studies were conducted in multiple locations, globally (Tables 4–6). Duration of interventions ranged from four weeks to six months. Both crossover studies were 8 weeks with a 2-week washout period.^(16,27)^ Intervention delivery varied across different studies. Six of the studies provided some or all of the food items within the diet intervention.^(16,20,23,26–28)^ One cross-over study by Fritsch et al. provided participants with all meals based on menus created by a Registered Dietitian (RD).^(27)^ Twelve of the included studies provided in-person or personalized diet education from an RD or nutritionist on how to follow the designated diet.^(14,16–25,28)^ Additionally, two studies had access to online support while following the diets.^(21,25)^ Alternatively, three studies provided diet education via handouts or other written material.^(15,26)^

**Table 4.** Characteristics and results of studies using elimination diets to improve quality of life among participants with IBD

Author, y	Country	Design/blinding	Duration	Population (male/female)	IBD type	Diet	QOL	Main findings
Cox et al., 2020^18^	London, England	Randomized, parallel, single-blinded, placebo control Participant blinded to diet	4 weeks	52 adult patients from gastroenterology clinics (23/29)	CD or UC	Intervention: Low FODMAP diet (*n* = 27) Control: sham diet (*n* = 25)	IBDQ	The Low-FODMAP diet group experienced significant improvements in QOL when compared to the sham diet group; (*P* = 0.042). The Bowel II domain score of the IBDQ was significantly higher in the low FODMAP diet group compared to the sham diet group; (*P* = 0.031).
Pederson et al., 2017^17^	Denmark	Randomized controlled, open-label	6 weeks	89 adult patients from tertiary hospital (22/67)	CD and UC	Intervention: Low FODMAP (*n* = 44) Control: Normal diet (*n* = 45)	SIBDQ	The Low FODMAP diet group had significant improvements in median (IQR) QOL scores from baseline (47; 42–55) to 6 weeks (60; 51–65, *p* < 0.01); however, the normal diet group did not from baseline (50; 40–57) to 6 weeks (50; 39–60, *P* = 0.54). When comparing the changes between the groups at 6 weeks the Low FODMAP diet group had significantly greater changes in QOL scores than the normal diet group, (*P* < 0.01).
Bodini et al., 2019^14^	Genoa, Italy	RCT	6 weeks	55 patients from a gastroenterology institution (24/31)	CD or UC	Intervention: Low FODMAP diet (*n* = 26) Control: Standard diet (*n* = 29)	IBDQ	The Low FODMAP diet group had significant improvements in median (IQR) IBDQ scores (baseline 166 (IQR 139–182) to follow-up 177; 155–188, *P* = 0.05). The standard diet did not. Baseline (181; IQR 153–197) to 166; IQR 153–200, *P* =(0.880). However, there was no significant difference found between the groups, (*P* = 0.886) at the end of 6 weeks.
Melgaard et al., 2022^16^	Denmark	Three group, double-blind, RCT, open-label, cross-over (feasibility only)	8 weeks	19 adult patients from gastroenterology clinics (2/17)	UC in remission with comorbid IBS	Control group: watchful waiting (*n* = 7) Intervention group (Phase 2): Low FODMAP with FODMAP provocation followed by placebo (*n* = 12) Intervention group (phase 3): FODMAP with placebo provocation followed by FODMAP provocation (*n* = 12)	SF-36	Within the low FODMAP group, there was median (IQR) increase in QOL score of 3.2 (1.1; 7.5) after FODMAP provocation and 2.2 (−3.5;4.3) after placebo provocation. However, there was no significant difference in QOL scores between the groups, *P* = 0.24. Additionally, there was a median (IQR) decrease in QOL scores for the control group of −5.7 (−10.3;0.3)
Jian et al., 2018^19^	Tianjin, China	Open label, stratified prospective	6 months	97 patients from Tianjin Medical University Hospital (50/47)	UC	Intervention: Food exclusion diet group (*n* = 49) Control: Sham diet (*n* = 48)	IBDQ	Specific IBDQ scores were not provided by the authors in the figures. It was noted that the food exclusion group had significantly greater QOL scores when compared to the control group in each dimension – bowel systems, systematic systems, emotion function (*P* < 0.05) and social function (*P* < 0.01). No comparison of changes between groups were made.
Gunasekeera et al., 2016^15^	London, England	Double-blinded, sham controlled, RCT	4 weeks	76 patients from three hospitals (32/44)	CD	Intervention: True diet (IgG4 diet) (*n* = 39) Control: Sham diet (*n* = 37)	SIBDQ	The True diet group had a 5.78 mean increase in QOL score, whereas the Sham diet group had a 1.07 mean increase in QOL scores. When adjusting for baseline and comparing between groups, the True diet group was significantly greater than the control in the intention to treat (*P* = 0.05) and per protocol (*P* = 0.007). The True diet group had scores that were 3.05 points better than the Sham diet group.
Bartel et al., 2008^20^	Vienna, Austria	Double-blinded, RCT	6 weeks	14 patients from Medical University of Vienna and General Hospital of Vienna (9/5)	CD	Intervention: Active group (organic diet) (*n* = 5) Control: low-fat, high carbohydrate diet (*n* = 9)	IBDQ	There was no significant difference in mean (SD) IBDQ scores in the active group from baseline (182, 23) to week 6 (196, 20; *P* = 0.204). Similarly, there was no significant difference in mean (SD) IBDQ scores in the control group from baseline (180, 15) to week 6 (192, 20; *P* = 0.071) The IBDQ scores were not compared between the groups.

CD, Crohn’s disease.

UC, Ulcerative colitis.

IBS, irritable bowel syndrome.

RCT, randomized controlled trial.

Short-form 36 (SF-36), An increase in the questionnaire score indicates an increase in Quality of Life (QOL).

Inflammatory Bowel Disease Questionnaire (IBDQ), The bowel II domain score of the IBDQ, measures GI symptoms impact on QOL.

Short Inflammatory Bowel Disease Questionnaire (SIBDQ), An increase in the questionnaire score indicates an increase in QOL.

**Table 5.** Characteristics and results of studies using an elimination diet that also increased specific foods among participants with IBD

Author, y	Country	Design/blinding	Duration	Population	IBD Type	Diet	QOL	Main findings
Tamizifar et al., 2020^23^	Isfahan, Iran	Parallel-group, single blind, RCT	12 weeks	76 Adult outpatients with confirmed diagnosis of UC (42/34)	UC	Intervention: DMP – low fat and simple carbs, low FODMAP, eliminate dairy (if lactose intolerant), alcohol and caffeine (*n* = 40) Control: Routine dietary advice (UDA) (*n* = 40)	SF-36	The DMP group had significant increases in QOL in three of the eight scales of the SF-36, represented as mean±SD. The control group had significant decreases in QOL in three of the eight scales of the SF-36. There were significantly greater improvements in the DMP group when comparing to the Control group in the following sub-scales; role-physical (62.2 ±6.1 48.0 ± 3.4, *p* = 0.06; 80 ± 3.6 to 74.4 ± 3.3, *P* < 0.001; *P* = 0.04), bodily pain (55.4 ± 4.0 to 73.9, *P* < 0.0001; 78.0 ± 3.7 to 70-±4, *P* = 0.01; *P* = 0.008), and general health (46.8 ± 2.9 to 68.5 ± 1.2, *P* < 0.001; 60.0 ± 2.7 to 62.0 ± 2.6, *P* = 0.61; *P* = 0.004).
Kyaw et al., 2014^22^	United Kingdom	RCT	4–6 weeks on diet with 6 months follow-up	112 adult outpatients with confirmed diagnosis of UC (62/50)	UC	Intervention: DMF (low fat, low FODMAP, low fibre, high protein and use of probiotic) (*n* = 61) Control: Healthy eating handouts (*n* = 51)	IBDQ	IBDQ scores increased slightly in the DMF group (*P* = 0.13) and decreased in the control group (*P* = 0.21). There were no noted differences between groups.
Keshteli et al., 2022^24^	Alberta, Canada	Open-label, RCT, parallel-group study	6 months	53 UC patients in clinical remission (19/34)	UC	Intervention: AID (*n* = 26) Control: CFG (*n* = 27)	SIBDQ	There was no significant change in SIBDQ scores in AID group from baseline (5.5 ± 0.9) to the last visit (5.6 ± 0.8, *P* = 0.56) or in the control group from baseline (5.5 ± 0.7) to the last visit (5.5 ± 0.9, *P* = 0.80). SIBQ scores were not compared between groups.
Chandrasekaran et al., 2019^21^	California, US	Uncontrolled, open-label	11 weeks	15 Gastroenterology adult outpatients (4/11)	CD or UC	Intervention: AIP Diet – 6 week elimination phase followed by 5 week maintenance phase (avoiding sugar, gluten, grains, legumes, nightshades, dairy, eggs, coffee, nuts, seeds, and food additives) (*n* = 15)	SIBDQ	There was a significant increase in QOL from baseline to weeks three (*P* = 0.02), six (*P* = 0.02), and nine (*P* = 0.03). There was no significant increase found in QOL at week 11 (*P* = 0.07). There was no control group to compare whether the intervention was better than a control.

CD, Crohn’s disease.

UC, ulcerative colitis.

RCT, randomized controlled trial.

DMP, Dietary Modification Program.

DMF, Dietary Modification Framework.

Short-form 36 (SF-36), An increase in the questionnaire score indicates an increase in Quality of Life (QOL).

Inflammatory Bowel Disease Questionnaire (IBDQ), The bowel II domain score of the IBDQ, measures GI symptoms impact on QOL.

Short Inflammatory Bowel Disease Questionnaire (SIBDQ), An increase in the questionnaire score indicates an increase in QOL.

**Table 6.** Characteristics and results of studies using dietary interventions that focus on increasing specific foods among participants with IBD

Author, y	Country	Design/blinding	Duration	Population	IBD Type	Diet	QOL	Main findings
Fritsch et al., 2021^27^	Florida, US	Randomized, Parallel-group, cross-over	8 weeks	18 adult patients from a Crohn’s and Colitis Clinic (7/11)	UC	Intervention: Low-Fat, High-Fibre Diet (*n* = 18) Control: improved Standard American Diet (iSAD) (*n* = 18)	SIBDQ	QOL improved significantly for both the intervention and control groups; *P* = 0.001, *P* = 0.02, respectively. Improvements in scores were significantly better for the low-fat, high fibre diet, *P* = 0.04.
Brotherton et al., 2014^26^	Virginia, US	Randomized controlled, single-blind	4 weeks	7 adult outpatients from an IBD clinic (3/4)	CD	Intervention: High Fibre (*n* = 4) Control: Attention control (*n* = 3)	IBDQ	The intervention group had significantly greater increases in QOL when compared to the control group, *P* = 0.028.
Lewis et al., 2021^25^	Multiple sites across the US	Parallel RCT	12 weeks	191 Patients from multi-site clinics (70/121)	CD	Intervention: Mediterranean Diet (*n* = 92) Control: Special Carbohydrate Diet (*n* = 99)	SIBDQ	There was a significant increase in QOL in both the Mediterranean diet group and the SCD group from baseline to week 6, *P* < 0.0001 for both groups. The changes in QOL for both groups at week 12 was not noted. There was no significant difference between the groups QOL at week 6 or week 12, *P* = 0.55 and *P* = 0.4, respectively.
Chicco et al., 2021^28^	Cagliari, Italy	Prospective intervention, uncontrolled	6 months	142 gastroenterology adult outpatients (76/66)	CD or UC	Intervention: Mediterranean Diet (*n* = 142)	IBDQ	In both groups of IBD types, QOL scores had significantly improved, *P* < 0.0001 for both groups.

CD, Crohn’s disease.

UC, ulcerative colitis.

RCT, randomized controlled trial.

Short-form 36 (SF-36), An increase in the questionnaire score indicates an increase in Quality of Life (QOL).

Inflammatory Bowel Disease Questionnaire (IBDQ), The bowel II domain score of the IBDQ, measures GI symptoms impact on QOL.

Short Inflammatory Bowel Disease Questionnaire (SIBDQ), An increase in the questionnaire score indicates an increase in QOL.

### Participant characteristics

Both sexes were included in all studies; however, more than half of the population studied were females. All studies included adult participants, 18 years and older, with a diagnosis of IBD. The IBD diagnosis was separated into subtypes: CD or UC. Where some studies included both subtypes,^(14,17,18,21,28)^ most studies did not. Crohn’s disease was studied independently within four studies, including the highly restricted organic diet, IgG4 diet, Mediterranean Diet, and high-fibre diet.^(15,20,25,26)^ Ulcerative colitis was studied independently in six studies including the low FODMAP diet, IgG diet, Dietary Modification Program, Dietary Modified Framework, and Anti-inflammatory diet interventions.^(16,19,22,23,24,27)^

Among the disease types, disease activity was assessed as an inclusion/exclusion criteria for each study. Most studies only included those in remission or with mild to moderate symptoms as defined by a disease severity tool (Table 1). Depending on IBD type, researchers used appropriate screening tools to select participants in either remission or with mild to moderate to severe symptoms. Tools used to screen for CD included the Harvey Bradshaw Index,^(4,14,15,17,18,21)^ partial Harvey Bradshaw Index,^(26)^ and the Crohn’s Disease Activity Index.^(6,15,20,28)^ The Mayo Score,^(14,16,19,21,27)^ Partial Mayo score,^(24)^ and Simple Clinical Colitis Index^(17)^ were used to screen and classify participants with UC.

### Medications and supplements

Treatments for IBD may include surgeries and medications all of which can be very costly and have serious or unwanted side effects.^(2,9)^ Medications are the most commonly used therapy when treating symptoms of IBD. The typical types include biologics, immunomodulators, aminosalicylate (5-ASA), corticosteroids, anti-TNF, and azathioprine.^(2)^ Each study had different, specific inclusion and exclusion criteria for medication type, dosage, and time frame of use. Researchers in 14 of the 15 studies allowed medication use as a concomitant treatment yet required that participants remain on a stable dose throughout the study duration.^(14–18,20–28)^ Jian et al. did not report use of medication amongst participants, but they did report that changes in medical treatment would be considered for each participant.^(19)^ Although supplements such as vitamins, minerals, and probiotics may affect QOL in this population, only four studies discussed the use of supplements as inclusion and exclusion criteria. Cox et al.^(18)^ and Tamizifar et al.^(23)^ excluded those who used or changed the dose of probiotics or prebiotics 8 weeks prior to the start of the study. Fritsch et al.^(27)^ excluded individuals who had used probiotics 4 weeks prior to the start of the study, whereas Bartel et al.^(20)^ excluded anyone who had used probiotics. The remaining authors did not mention the use of supplements as an inclusion or exclusion criterion.

### Changes in quality of life after following an elimination diet

Four studies used the low FODMAP diet for the intervention (Table 4).^(14,16–18)^. Two of the four studies where the low FODMAP diet was compared to a sham or usual diet found significantly better and positive changes in QOL when compared to the control.^(17,18)^ In a four-week randomized, parallel, single-blinded placebo control study, Cox et al. provided diet advice from an RD to 52 participants with either UC or CD.^(18)^ At follow-up, the low FODMAP group experienced significantly greater IBDQ scores (81.9, SEM 1.2) compared to the sham diet group (78.3, SEM 1.2, *P* = 0.04). Pederson et al. also had nutritionists provide a one-hour counselling session on FODMAPs to the experimental group while the control group was asked to remain on the normal diet. After six weeks, the group following the low FODMAP intervention had a significantly greater increase in SIBDQ scores reported as median and interquartile ranges (IQR) compared to the normal diet group, (60, IQR 51–65 and 50, IQR 39–60, *P* < 0.01).^(17)^ Additionally, scores were compared within groups from baseline to the end of the study, where the low FODMAP group had a significant increase in median scores (47, IQR 42–55 and 60, IQR 51 to 65, *P* < 0.01), but the normal diet group did not (50, IQR 40–57 and 50, IQR 39–60, *P* = 0.54), respectively. Similar to Pederson, Bodini et al. utilized a six-week low FODMAP diet intervention compared to a Standard Diet (SD) to evaluate changes in QOL.^(14)^ There was no significant difference between the scores reported as median and IQR from baseline to 6 weeks in the SD group (181, IQR 153–197 and 166, IQR 153–200, *P* = 0.88, respectively). However, there was a slight but significant increase in median survey scores in the low FODMAP diet group from baseline to 6 weeks (166, IQR 139–182; 177, IQR 155–188, *P* = 0.05, respectively). A positive outcome cannot be concluded as there was no significant difference found between groups at the end of 6 weeks (*P* = 0.88).

Lastly, in a three group, double blind RCT crossover study to test feasibility, Melgaard et al. examined changes in QOL in 19 adults with UC after completing three phases, including watchful waiting, low FODMAP with FODMAP provocation, and placebo provocation.^(16)^ Provocation arms were used to provide participants with food containing FODMAPs that appeared and tasted similar to foods with low-FODMAPs. These provocation foods were made in the lab and provided to participants. They found no significant differences in changes in QOL scores over 4 weeks between the low FODMAP and placebo provocation groups, respectively (3.2, IQR 1.1–7.5; 2.2, IQR −3.5–4.3, *P* = 0.24). Additionally, the watchful waiting group had a decrease in QOL scores after 4 weeks although no significance level was provided (−5.7, IQR −10.3–0.3). There was no significant difference found between the groups and therefore a positive outcome cannot be concluded (*P* = 0.24).

Another elimination diet to consider is the IgG diet. Using a randomized, open label, stratified prospective study, Jian et al. recruited individuals previously admitted to a local hospital.^(19)^ Participants were assigned to follow the IgG diet intervention or the sham diet. Researchers examined changes in IBDQ scores in 97 participants with UC over a 6-month IgG diet intervention compared to a sham diet. There was no significant difference in the mean IBDQ scores between the groups at baseline (*P* > 0.05). Overall, the intervention group had significantly higher IBDQ scores compared to the sham diet group in all four dimensions; bowel symptoms, systemic symptoms, emotional function dimensions (*P* < 0.05), and the social function dimension (*P* < 0.01) after the 6-months. Similarly, Gunasekeera et al. studied an IgG4 diet, a subclass of the IgG diet, except only in those with CD.^(15)^ Individuals in the intervention group, known as the True diet, were to exclude the four foods with the highest titre response, indicating a greater immune response to those foods. The control group was instructed to exclude the four foods with the lowest titre response. Significant improvements were found in the intervention group when compared to the control after adjusting for baseline in both intention to treat and per protocol analyses, respectively (−3.05; IQR −6.11 to −0.01, *P* = 0.05 and −4.728; IQR −8.10 to −1.36, *P* = 0.007). Refer to Table 4 for details on the study results.

While the previously mentioned diets are commonly used elimination diets, Bartel et al. used a highly restricted organic diet (Table 2) in a double-blind randomized controlled trial.^(20)^ Eighteen participants with mild-to-moderate CD were randomized to the active group (organic diet) or control group for six weeks with a follow-up at 24-weeks. The control group was instructed to eat a low-fat, high-carbohydrate diet while avoiding red meat and fibrous fruits and vegetables. Both groups received nutrition counselling by an RD at weeks 1, 3, and 6 of the intervention and weeks 12 and 24 of the follow-up period. The active group did not experience a significant change in IBDQ scores (reported as mean ± SD) from baseline to week 6, respectively (182 ± 23 and 196 ± 20; *P* = 0.204). Similarly, the control group did not experience a significant change in IBDQ scores from baseline to week 6 (180 ± 15 and 192 ± 20; *P* = 0.071). The changes in IBDQ scores were not compared between the intervention and control groups.

### Changes in quality of life after following an elimination diet with increased specific foods

Research teams for four studies used an elimination diet paired with the addition of specific foods or nutrients (Table 5).^(21–23)^ In two of the four studies, research teams created similar dietary programmes for UC; however, only one found significant improvements in QOL following the intervention.^(22,23)^ Tamizifar et al. created a 3-month Dietary Modification Program intervention (Table 2) for adults with UC, provided by RD instruction and an educational booklet.^(23)^ The control group received usual dietary advice (UDA). After adjusting for differences in baseline data, when compared to the control group, the Dietary Modification Program group had significantly greater improvements in three of the eight scales within the SF-36, including role-physical, bodily pain, and general health. Similarly, Kyaw et al. utilized the Dietary Modified Framework (Table 2) intervention in adults with UC.^(22)^ The diet advice was provided through an educational booklet over four to six weeks. Participants in the control group received healthy eating handouts. QOL was measured using the IBDQ, which was noted to increase slightly in the Dietary Modified Framework group with a mean difference of 7.173, yet this was not a significant change (*P* = 0.13). The control group was noted to have a decrease QOL scores with a mean difference of 3.454; however, this change was also not considered significant (*P* = 0.21). Between group changes were not noted.

In an open-label, randomized controlled, parallel study, Ketshteli et al. conducted a 6-month dietary intervention utilizing the Anti-Inflammatory Diet versus a control diet in adults with UC.^(24)^ Twenty-six participants randomized to the Anti-Inflammatory Diet group received dietary counselling, including menus and recipes, once a month from an RD. Twenty-seven participants randomized to the control group were instructed to follow the Canada Food Guide (CFG) 2007.^(34)^ There was no significant difference in SIBDQ scores reported as mean ± SD between the Anti-Inflammatory Diet group and the CFG group at baseline (5.5 ± 4.9–6.4 and 5.0 ± 5.0–6.0; *P* = 0.99). Furthermore, there was no significant difference in SIBDQ scores in the Anti-Inflammatory Diet group from baseline (5.5 ± 0.9) to the last visit (5.6 ± 0.8, *P* = 0.50) or in the CFG group from baseline (5.5 ± 0.7) to the last visit (5.5 ± 0.9, *P* = 0.80). There were no results reporting changes between the groups.

The Autoimmune Protocol Diet is an established diet used to reduce symptoms in autoimmune diseases. In an uncontrolled, open label study, Chandrasekaran et al. examined changes in QOL using the SIBDQ in 15 CD or UC participants after following the Autoimmune Protocol Diet over 12-weeks in an uncontrolled trial.^(21)^ SIBDQ mean scores increased steadily at all time periods of the intervention as follows: baseline (46.5; SD 12.5) to week 3 (54.0; SD 7.7, *P* = 0.02); week 6 (53.3 SD 10.9, *P* = 0.02); and week 9 (62.0; SD 3.3, *P* = 0.03). However, from all participants that completed follow-up measures (*n* = 4), there was not a significant increase in SIBDQ scores from baseline to week 11 (46.5 to 61.5, *P* = 0.07). Differences in SIBDQ scores between CD and UC were also explored and there was no significant difference found between the IBD types (*P* = 0.77). Finally, there was no control group for comparison in this study. Refer to Table 5 for details on the study results.

### Changes in quality of life while following dietary patterns with a focus on including specific foods

Four studies used dietary pattern interventions with a focus on including specific foods, such as the Mediterranean Diet or a high-fibre diet, to examine changes in QOL.^(25–28)^ Control diets for three of the four studies varied, and one study was uncontrolled. All four studies had significant improvements in QOL following the intervention diets; however, not all results were compared between groups. Fritsch et al. used a randomized, parallel-group cross-over design in 18 adults with UC to examine changes in SIBQ scores following a low-fat, high-fibre diet (Table 6) and an improved standard American diet (iSAD) as the control.^(27)^ All meals were provided to the participants. SIBDQ scores increased significantly in both the intervention (*P* = 0.001) and control groups (*P* = 0.02) after 4 weeks on each diet. There were significant improvements in many of the scales within the SF-36 within both groups, including limitations due to physical health scores, role limitations due to emotional problems scores, social functioning scores, and bodily pain scores. Overall, the general health scores significantly improved from a baseline average of 48.5 in both the intervention and the control (59.1, *P* = 0.03 and 57.6, *P* = 0.04).

Similar to Fritsch, Brotherton et al. examined changes in QOL using a high fibre diet.^(26)^ However, this was a randomized controlled, single-blinded study that used an attention control diet in 7 adults with CD over 4 weeks. The high fibre diet was not defined by the researchers; however, the intervention group received education on high-fibre, low-refined carbohydrate intake, and were encouraged to consume ½ cup of provided, whole wheat bran cereal and at least 48 oz of fluid per day, whereas the control group was instructed to avoid whole grains. They found that IBDQ scores significantly increased over time for the intervention group when compared to the control group, (*P* = 0.028). Clinical significance, defined by authors as a > 32-point increase, was also found in the high fibre diet group with a mean increase of 44.25-points in IBDQ scores whereas the control group had a mean increase of 19-points. This study collected additional data on diet adherence but did not report the findings. The small sample size of 7 should be considered when interpreting the results of this study.

Two studies explored changes in QOL after following the Mediterranean diet (MD).^(25,28)^ However, Lewis et al.^(25)^ used the Specific Carbohydrate diet (SCD), a diet commonly used within IBD, as the control diet in a parallel RCT with 194 participants with CD, whereas Chicco et al.^(28)^ did not use a control diet and included 142 participants with either UC or CD. Lewis et al. found improvements in SIBDQ scores in the SCD group which was associated with an increase in standardized mean difference of SIBDQ scores from baseline to 6 weeks, (8.85 to 10.4, *P* < 0.0001); however, there was no report on the results from 6–12 weeks.^(25)^ Additionally, the SCD did not have a significantly greater increase in median SIBDQ scores at week 6, (49.0, 39.0–58.0) compared to MD median scores at week 6, (49.5, 39.0–56.0, *P* = 0.55). Similarly, the SCD did not have a significantly greater increase in median SIBDQ scores at week 12, (50.0, 38.0–58.0) compared to MD median scores, (45.5, 36.0-57.0, *P* = 0.040). Chicco et al. found significantly improved IBDQ scores after the intervention (*P* < 0.0001) yet did not have a control group for comparison between groups.^28^ Refer to Table 6 for additional details on study results.

## Discussion

Although this research is in its preliminary stages, it appears as if a wide range of dietary approaches have the potential to positively impact QOL in adults with IBD. Significant improvements in QOL were found in six of the 15 studies, including interventions using the low FODMAP,^(17,18)^ IgG,^(15,19)^ Dietary Modification Program,^(23)^ and high fibre diet.^(27)^ The Autoimmune Protocol,^(21)^ Anti-inflammatory Diet,^(24)^ highly restricted organic diet,^(20)^ Dietary Modified Framework,^(22)^ and Mediterranean Diet^(25,28)^ did not result in significantly improved QOL. Most of the dietary approaches were compared to a sham control diet or did not include a control, with one exception, where Lewis et al. compared the Mediterranean Diet to the Specific Carbohydrate Diet.^(25)^ Thus, superiority of one dietary approach over another cannot be determined at this time.

Our results are similar to others using different search terms. Gomes et al. focused on dietary intake and gut microbiota in adults with IBD.^(13)^ Similar to the findings of this review, Gomes et al. found that some of the same diets, including the MD, SCD, and low FODMAP diet may assist in reducing IBD symptoms, thus improving QOL.^(13)^ However, this research team only focused on those with UC in remission. Lemketkai et al. conducted a systematic review and meta-analysis using 27 RCT studies to examine dietary interventions as a treatment for IBD.^(10)^ Ten of the 27 studies measured QOL, of which six were included in this review. Although the purpose of their review was to focus on dietary interventions inducing remission, it was also noted that a diet high in fibre was not associated with reducing relapse despite increases in QOL in those with CD. Additionally, similar to this review, Limketkai et al. found that there were no improvements in QOL associated with the highly restricted organic diet or the AID. Furthermore, significant improvements in QOL were noted in other dietary intervention studies, such as a high-fibre diet, SCD, and MD diets. Yet only the high-fibre diet was found to have significant improvements in QOL when compared to the control group, whereas others were only compared within groups. The research team found that the MD and SCD may promote clinical remission for those with CD. However, there were no dietary interventions found to be associated with inducing remission in those with UC. Lemketkai et al. concluded a need for additional dietary intervention studies in IBD to increase the certainty of evidence that can help develop nutrition guidelines for IBD.^(10)^

Within all 15 studies reviewed, validated-specific questionnaires were used to assess health-related QOL. Despite these findings, it is difficult to determine if these diets should be recommended long-term as these studies had short study durations. Within the reviewed studies, three of the low FODMAP diet studies did not include the recommended reintroduction or maintenance phase of the diet. Thus, a conclusion cannot be drawn for the long-term impact of this diet. Elimination diets can be difficult to follow and could result in the restriction of foods if not followed correctly. Therefore, additional studies are needed to evaluate the long-term effects of these diets on QOL in addition to adequate nutrient intake. Dietary patterns such as the MD or high fibre diet may be more sustainable and easier to implement. Furthermore, the MD provides additional health benefits such as lowering the risk of cardiovascular disease and cancer, which are common comorbidities for those with IBD.^(7,25)^

The impact of these nine different diet interventions on QOL outcomes in adults with IBD requires further research. As noted, one limitation was the inclusion of only one subgroup, CD or UC. A reoccurring limitation amongst these studies is the small sample sizes; therefore, the reported changes in QOL were often underpowered. Additionally, most of these studies utilized a controlled sham diet that mimicked usual dietary intake rather than comparing dietary interventions. Thus, this design does not show the differences in changes in QOL across the different diet types, such as comparing an elimination dietary intervention to a dietary pattern intervention. However, these interventions ranged widely from elimination diets to inclusive, more sustainable diets. As mentioned previously, these dietary interventions were of short durations and did not always carry out all phases of the diets. Future research should focus on long-term benefits of dietary adherence. Additionally, the changes in QOL were not always reported clearly, making it difficult to draw conclusions. Because many of the study participants had mild to moderate symptoms, these diets cannot be generalized to all individuals with IBD.

Future research should compare the different dietary approaches and assess disease specific outcomes, diet adherence, adequacy of the diet long-term, sustainability of the diet long-term, other eating behaviours, and QOL. Similar to Lewis et al.^(25)^ that compared the MD and SCD, researchers may consider comparing some of these dietary approaches to one another in a single study. It may be easier to highlight a superior dietary approach when they are compared side by side. For example, while elimination diets may be helpful in identifying foods and beverages that may be associated with symptoms, these diets are typically not appealing due to strict restrictions, nor are they meant to be followed long-term.^(15)^ Therefore, it should be noted that QOL may increase temporarily while strictly following an elimination diet due to changes in symptoms. Additionally, more studies are needed that include adequate numbers of both groups of CD and UC to understand the impact of dietary interventions in both groups. Results from dietary approaches may differ between the subgroups due to the previously mentioned differences between the disease location and inflammation. Larger sample sizes and longer study durations should also be a priority for future researchers to improve certainty of evidence.

Overall, dietary patterns that focus on specific foods or food groups showed the most consistent results, leading to increased QOL after the intervention diets. This is likely because they are more sustainable. Elimination diets, particularly the low FODMAP diet, showed mixed outcomes, with two studies reporting significant QOL improvements, likely due to the support from Registered Dietitians (RD) and nutrition counselling, which can improve sustainability. Conversely, studies with elimination diets that included specific foods had the fewest significant QOL increases after dietary changes, resulting in less consistent evidence.

Finally, the diversity of study designs and interventions can affect the interpretation of QOL outcomes when comparing studies. For example, in RCTs, the cross-over design may yield more robust results than double-or single-blinded designs because it allows participants to serve as their own controls. Similarly, the RCT studies will yield more reliable results than the uncontrolled trials because they allow comparison with a control group. Those with longer study durations demonstrate the sustainability of the dietary interventions and their long-term impact on QOL. Additionally, participants who received meals may have higher participation and lower attrition than those who did not, thereby influencing QOL outcomes.

## Conclusion

### Implications for practice

This review highlights the potential for several dietary interventions to positively impact QOL in adults with IBD. These diets are complex and require nutrition counselling from an RD. In 12 of the 15 studies, an RD was used to help explain the diet and counsel participants.^(14,16–21,23–25,27,28)^ For example, the low FODMAP diet should be carried out in three phases as opposed to remaining in the elimination phase long-term. Additionally, a high fibre diet is contraindicated for individuals with strictures. Therefore, an RD with experience in the treatment of IBD needs to be included as a member of the healthcare team to individualize diets to (1) reduce symptoms, (2) meet nutrient needs, and (3) accommodate preferences, resources, abilities, and lifestyles for adults with IBD. Based on the results from this study, these diets are likely most appropriate for adults with IBD in remission or with mild to moderate symptoms.
